# Flagella are required to activate expression of aggregation factors necessary for T6SS-mediated competition in host-like conditions

**DOI:** 10.1128/mbio.03970-24

**Published:** 2026-08-27

**Authors:** Lauren Speare, Liang Zhao, Morgan N. Pavelsky, Aundre Jackson, Steph Smith, Bhavyaa Tyagi, Garrett C. Sharpe, Madison Woo, Scott M. Gifford, Alecia N. Septer

**Affiliations:** 1 School of Biological Sciences, Georgia Institute of Technology 1372, Atlanta, Georgia, USA; 2 Department of Earth, Marine & Environmental Sciences, University of North Carolina 311956, Chapel Hill, North Carolina, USA; Indiana University Bloomington, Bloomington, Indiana, USA

**Keywords:** *Aliivibrio fischeri*, viscosity, transcriptomics, type VI secretion, aggregation

## Abstract

Bacteria employ antagonistic strategies to eliminate competitors of an ecological niche. Contact-dependent mechanisms, such as the type VI secretion system (T6SS), are prevalent in host-associated bacteria, yet we know relatively little about how T6SS+ strains make contact with competitors in highly viscous environments, such as host mucus. To better understand how cells respond to and contact one another in such environments, we performed a genome-wide transposon mutant screen of the T6SS-wielding beneficial bacterial symbiont, *Vibrio fischeri* MJ11, and identified two sets of genes that are conditionally required for killing. We found that surface modification and flagellar-associated genes do not affect T6SS directly and are therefore not required for interbacterial killing when cell contact is forced, yet are necessary for killing in high-viscosity liquid (hydrogel), where cell-cell contact must be biologically mediated. Quantitative transcriptomics revealed that *V. fischeri* significantly increases expression of both T6SS genes and cell surface modification factors upon transition from low- to high-viscosity media. Consistent with coincubation and fluorescence microscopy data, flagella are not required for T6SS expression in hydrogel. However, flagella were necessary to enhance expression of ~50% of the genome in hydrogel, including the surface modification genes identified in our screen and functional pathways important for host colonization, such as uptake of host-relevant iron and carbon sources, and nitric oxide detoxification enzymes. Our findings suggest that flagella play a key role when *V. fischeri* cells coordinately activate competitive strategies and host colonization factors, underscoring the significance of the physical environment in directing complex bacterial behaviors.

**IMPORTANCE:**

The physical environment has dramatic effects on bacterial behavior, but little is known about how mechanical signals impact antagonistic interactions. Symbiotic bacteria use molecular weapons to eliminate competitors for limited space within highly viscous host tissue and mucus. Previously, we found that a putative lipoprotein adhesin, TasL, and an unknown ligand are required to bring competitor cells within range of the T6SS weapon. Here, we found that mutations in flagella or predicted surface modification genes prevent TasL-mediated adhesion and killing in high viscosity. Transcriptomics revealed the flagella are required to coordinate expression of host colonization factors with the T6SS interbacterial weapon when transitioning from lower to higher viscosity conditions. These findings suggest that flagella may play a role in sensing mechanical signals, such as environmental viscosity, to enhance a symbiont’s ability to successfully colonize the host while efficiently eliminating potential competitors from the host niche.

## INTRODUCTION

Bacterial competition is prevalent in free-living and host-associated communities, and a myriad of competitive strategies have been identified (1). Many of these mechanisms require direct cell-cell contact for inhibitor cells to deliver effector proteins directly into target cells (2). On surfaces, bacteria often grow in dense communities that facilitate this necessary contact. However, little is known about how bacteria make contact in liquid environments. The type VI secretion system (T6SS) is a contact-dependent mechanism that allows inhibitor cells to deliver effector proteins directly into target cells through a syringe-like apparatus. T6SSs are prevalent in free-living, symbiotic, and pathogenic species (3, 4) and are active in animal microbiomes (5–10).

The bioluminescent squid and fish symbiont *Vibrio* (*Aliivibrio*) *fischeri* encodes a strain-specific T6SS (T6SS2) that allows cells to eliminate competitors during symbiosis establishment (5, 11). *V. fischeri* activates T6SS2 expression and killing upon transition from a low- to high-viscosity liquid environment, which mimics the increase in viscosity cells experience during host colonization (12). In high-viscosity liquid (hydrogel), *V. fischeri* makes large, mixed-strain aggregates that are biologically mediated and pH-dependent; these aggregates facilitate the contact necessary for T6SS-mediated killing in a liquid environment (12, 13). We recently identified a putative cell-cell adhesion gene associated with T6SS2, *tasL,* that is required for target specificity and inhibitor-target contact in hydrogel via a heterotypic interaction with an unknown ligand (11). In addition to viscosity, the presence of calcium is also sufficient to activate T6SS2 expression and aggregation in low-viscosity liquid (14). However, the factors *V. fischeri* cells use to sense and respond to changes in environmental viscosity and promote cell-cell contact for T6SS2-dependent competition require further investigation.

Here, we sought to identify such factors by performing an unbiased screen to identify genes required for killing in high-viscosity liquid conditions. We screened transposon mutants of the T6SS2-encoding *V. fischeri* strain MJ11 and identified mutants that cannot kill a target in hydrogel but are still lethal on surfaces, suggesting they are unable to kill under conditions that require biologically mediated cell-cell contact. We characterized the impact of these “conditional” mutations, including flagellar mutants, on T6SS sheath assembly, aggregation, and motility. We then used quantitative transcriptomics to explore the role of flagella in regulating gene expression during habitat transition. The quantitative transcriptomic data generated from this analysis reveal the significant transcriptional shift *V. fischeri* cells undergo during habitat transition, the contribution of flagella in responding to changes in environmental viscosity, and insight into how a model beneficial bacterial symbiont coordinates activation of key competitive and colonization pathways in response to its physical environment.

## RESULTS

### Transposon mutagenesis of MJ11 identifies genes required for killing in hydrogel

**Fig 1:**
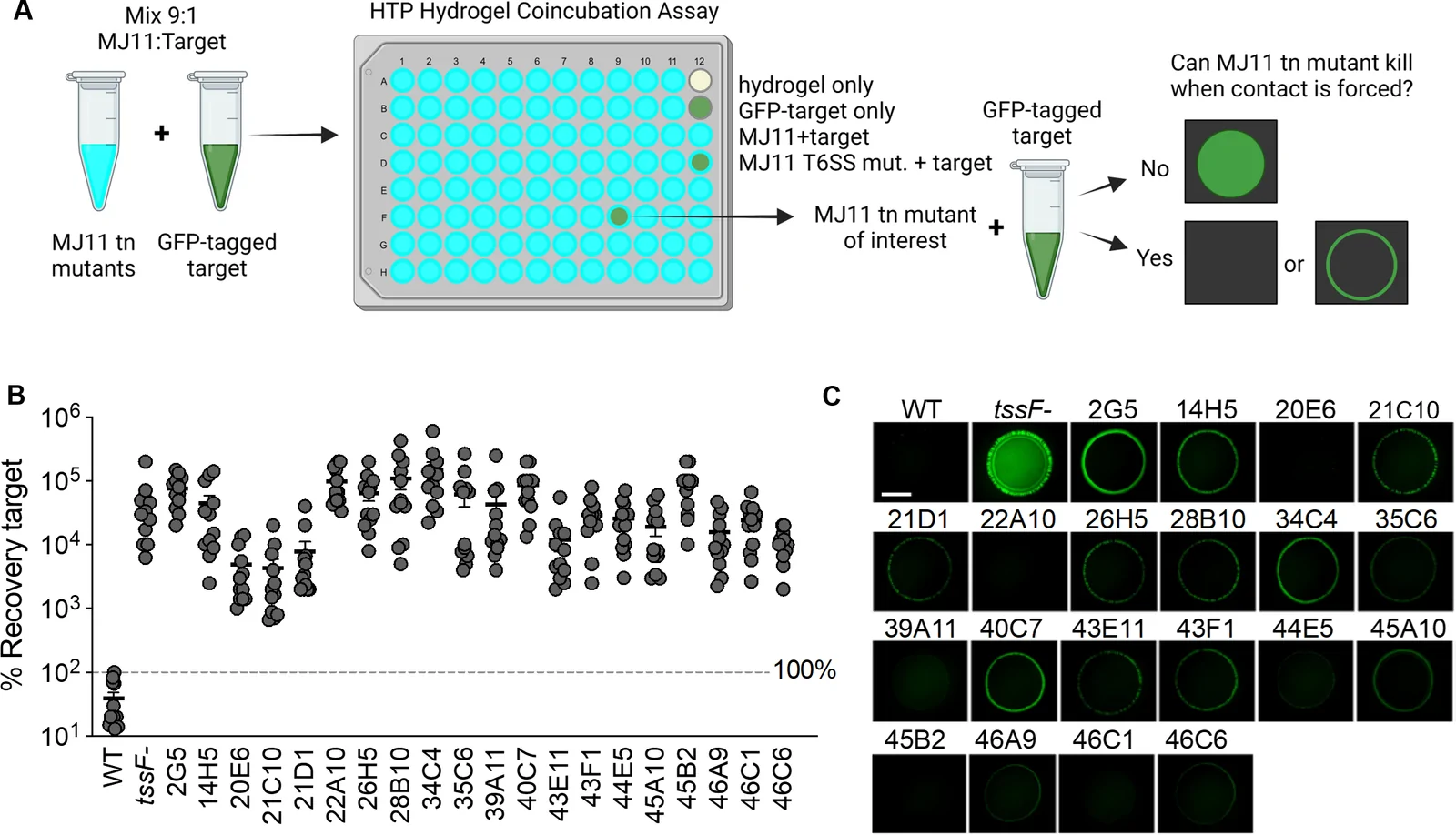
Transposon screen identified 20 conditional mutants. (**A**) Approach for initial screen to identify nonlethal MJ11 Tn mutants in hydrogel (made with Biorender.com). Tn mutants were arrayed into a 96-well plate containing LBS hydrogel medium and a culture of GFP-tagged ES114; cultures were mixed in a 9:1 MJ11:ES114 ratio. After 24 h, each well was assessed for luminescence and GFP to determine the presence of MJ11 and ES114, respectively. Wells with luminescence and fluorescence values similar to the target + MJ11 T6SS− control well were selected for future experiments. (**B**) Results of coincubation assays in hydrogel between MJ11 wild type (WT), T6SS2 disruption mutant (*tssF*−), or transposon mutant and ES114 (target) displayed as percent recovery of the target strain. MJ11 strain genotype is listed on the *x*-axis; transposon mutant names are abbreviated as follows: LAS2G5 shown as 2G5. Dashed line at 100% recovery indicates no net change in target growth over the 12-h coincubation period. (**C**) Results of coincubation assays on agar surfaces between MJ11 strains and ES114 shown as fluorescence microscopy images of ES114 taken at 24 h; scale bar = 2 mm. MJ11 strain name is shown above each coincubation spot. Each experiment was performed three times, and either combined data (B, *n* = 12) or a representative replicate is shown (C, *n* = 1).

To begin identifying genetic factors required for target cell elimination in hydrogel, we generated transposon mutants of the lethal *Vibrio fischeri* strain MJ11 and then screened these mutants for the ability to prevent the growth of an unarmed target *V. fischeri* strain, ES114, in hydrogel and on surfaces. MJ11 encodes the strain-specific T6SS2 genomic island (5) and kills ES114 in a T6SS2-dependent manner (13). MJ11 has a fully sequenced genome, allowing for identification of transposon insertions throughout the genome (15), and importantly is brightly luminescent in culture, compared with ES114 (16), which allowed us to discriminate between MJ11 and GFP-tagged ES114 in coculture. We incubated GFP-tagged ES114 with wild-type MJ11 or a T6SS structural mutant (*tssF_2/vasA_2*) as controls for target killing and target survival, respectively (Fig. 1A). Briefly, each MJ11-derived strain was mixed with GFP-tagged ES114 at a 9:1 (MJ11:ES114) ratio by volume and incubated in hydrogel (liquid LBS [Luria Bertani with added salts] + 5% polyvinylpyrrolidone [PVP]) without shaking for 24 h. To screen many mutants simultaneously, coincubation assays were optimized for 96-well plates, as described previously (11), and competitive outcomes were determined by the relative fluorescence units (RFU) of the GFP-tagged target strain. Any mutant that was unable to reduce the target RFUs was then confirmed through additional coincubation assays in hydrogel where target strain colony-forming units (CFUs) were quantified at the beginning and end of each experiment (Fig. 1B) (12). We screened 7,300 mutants and identified 20 mutants that showed reduced killing of ES114 in hydrogel (Fig. 1B) but could still prevent the growth of GFP-tagged ES114 when cell-cell contact was forced on agar surfaces (Fig. 1C).

We used a combination of cloning and inverse PCR to determine the transposon insertion site for each conditional MJ11 mutant. The transposons disrupted 12 unique genes found only on chromosome I (Table 1). The majority of disrupted genes (9/12) were in a predicted capsule/LPS biosynthesis gene cluster that shows a high degree of variation across *V. fischeri* strains, with the most well-studied strain (ES114 [17–19]) encoding 29 unique genes not found in other analyzed strains (Fig. S1). Transposons also disrupted flagellar motility genes (*flgK* and *flgB*) and a putative deaminase/amidohydrolase (Table 1). Given that conditional genes are not required for killing on surfaces, we hypothesized that the conditional genes identified here are involved in mediating the necessary cell-cell contact for T6SS killing in hydrogel.

**TABLE 1:** Location of each transposon insertion in the MJ11 genome^*a*^

MJ11 mutant strain name(s)	Gene ID	Annotation	Predicted pathway	Disruption location	Gene length
LAS34C4	VFMJ11_0136	O-antigen polymerase/ligase	Cell surface modification	521 bp	1,338 bp
LAS40C7	VFMJ11_0138	Capsular polysaccharide biosynthesis protein	Cell surface modification	67 bp	1,578 bp
LAS46C1 (1) LAS45A10 (2) LAS46C6 (3) LAS2G5 (4)	VFMJ11_0140	Lipopolysaccharide synthesis	Cell surface modification	68 bp (1) 343 bp (2) 430 bp (3) 772 bp (4)	963 bp
LAS14H5	VFMJ11_0174	UDP-2, 4-diacetamido-2,4,6-trideoxy-beta-L-altropyranose hydrolase	Cell surface modification	1,017 bp	1,503 bp
LAS44E5	VFMJ11_0175	Pseudaminic acid synthase	Cell surface modification	958 bp	1,062 bp
LAS26H5 (1) LAS28B10 (2)	VFMJ11_0179	LPS biosynthesis protein	Cell surface modification	142 bp (1) 447 bp (2)	1,179 bp
LAS43F1	VFMJ11_0180	Hypothetical protein	Cell surface modification	503 bp	1,359 bp
LAS43E11 (1) LAS21C10 (2) LAS35C6 (3) LAS46A9 (4) LAS39A11 (5)	VFMJ11_0181	Lipopolysaccharide flippase	Cell surface modification	466 bp (1) 531 bp (2) 702 bp (3) 709 bp (4) 718 bp (5)	1,143 bp
LAS21D1	Promoter of VFMJ11_0184	UDP-glucuronate 5’-epimerase	Cell surface modification	1 bp upstream ATG	1,005 bp
LAS20E6	VFMJ11_1236	Deaminase/amidohydrolase	Cysteine and methionine metabolism	908 bp	1,410 bp
LAS22A10	VFMJ11_2000	Flagellar hook-associated protein FlgK	Modify	59 bp	1,881 bp
LAS45B2	VFMJ11_2009	Flagellar basal body rod protein FlgB	Modify	264 bp	396 bp

^*a*^ Annotation and predicted pathways provided by KEGG.

### Conditional genes are not required for T6SS2 sheath assembly

**Fig 2:**
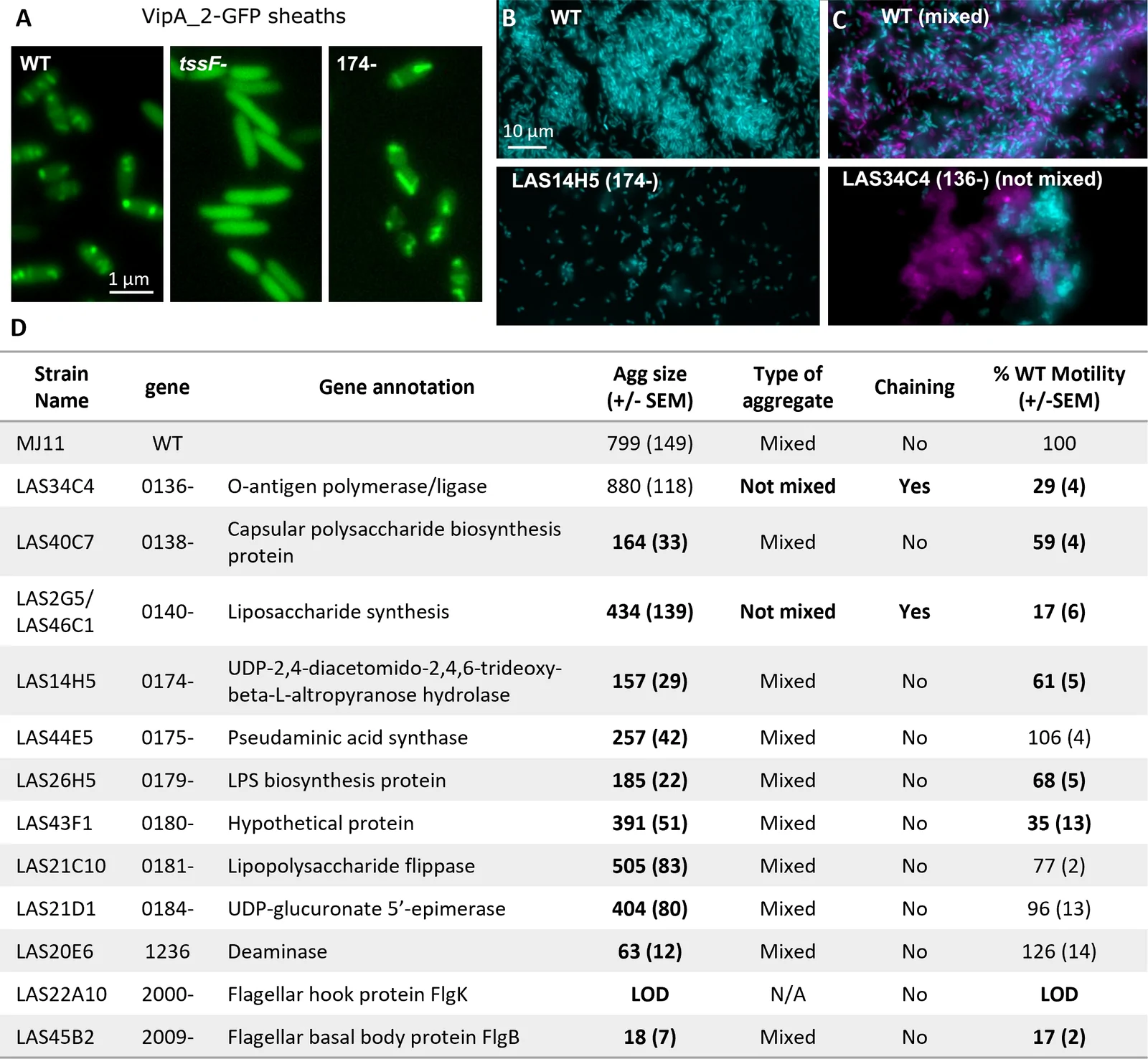
Motility and LPS/capsule genes are necessary for mixed-strain aggregates in hydrogel. (**A**) Representative fluorescence microscopy images of MJ11 WT, *tssF−,* or 174− harboring an IPTG-inducible VipA_2-GFP expression vector. Cultures were incubated in hydrogel supplemented with 1.0 mM IPTG; scale bar = 1 um. Sheaths were observed in all transposon mutants. Each experiment was performed twice with two biological replicates and two to three fields of view (*n* = 10). (**B and C**) Representative single-cell fluorescent microscopy images of (**B**) MJ11 monocultures or (**C**) differentially tagged but otherwise isogenic MJ11 cocultures incubated in hydrogel for 12 h; scale bar = 10 um. (**D**) Table displays results from experiments with representative transposon mutants where column 1 shows the strain name, column 2 indicates the disrupted gene, and column 3 shows the annotation for each gene. Columns 4–7 display results from the following experiments: (4) monoculture assays quantifying aggregation (Agg) size (cells per aggregate) after 12 h in hydrogel; (5) coculture assays with differentially tagged but otherwise isogenic MJ11 strains to determine whether aggregates were evenly mixed (C, top) or not mixed (C, bottom); (6) visual observation of whether cells displayed chaining in hydrogel; and (7) motility assays in LBS motility agar reported as percent of the wild type. Each experiment was performed at least twice with two biological replicates and five fields of view per replicate (aggregation assays, chaining assays; *n* = 20) or three times with four biological replicates (motility assay; *n* = 12). SEM indicates standard error of the mean; LOD indicates limit of detection.

To begin characterizing the role of conditional genes in T6SS-mediated competition, we tested whether any conditional genes were necessary for constructing a T6SS sheath in hydrogel. VipA/TssB is a subunit of the T6SS sheath, and by tagging it with GFP, we can visualize sheath assembly in live cells (5). We moved a VipA_2-GFP expression vector into a representative transposon mutant for each disrupted conditional gene and used fluorescence microscopy to visualize sheath assembly. Wild-type MJ11 cells contained fully extended GFP-tagged sheaths, while the MJ11 *tssF_2* mutant control strain, which lacks the essential baseplate component, contained no sheaths, and only diffuse GFP was observed in these cells (Fig. 2A). All conditional mutants contained VipA_2-GFP sheaths (Fig. S2). This observation suggests that conditional genes are not necessary for T6SS2 sheath assembly and is consistent with our hypothesis that conditional genes are involved in mediating the cell-cell contact required for killing in hydrogel.

### Surface modification and motility mutants are impaired in aggregation in hydrogel

The majority of disrupted conditional genes fall into two predicted functional categories: surface modification (putative capsule/LPS biosynthesis genes) and flagellar motility (*flgK* and *flgB)* (Table 1). To test the hypothesis that conditional genes are required for the necessary cell-cell contact for T6SS2 killing in hydrogel, we assessed the ability of representative conditional mutants (one mutant per disrupted gene) to form wild-type-sized mixed-strain aggregates, because we previously showed that deficiencies in either of these factors would reduce cell-cell contact between inhibitor and target and result in impaired T6SS-mediated killing in hydrogel (11, 13). The majority of transposon mutants (11 out of 12) had a significantly smaller average estimated aggregate size compared with the wild type (Fig. 2B and D; Fig. S3). Ten of these 11 aggregation-deficient mutants formed mixed-strain aggregates with differentially tagged yet otherwise isogenic strains, with both strain types distributed throughout the aggregates (Fig. 2C and D; Fig. S4). Two mutants (disruptions in VFMJ11_0136 and VFMJ11_0140), however, were observed to chain in culture (Fig. S2, white arrows) and formed aggregates that were not well mixed but rather were composed of disparate clusters of each strain type (Fig. 2C; Fig. S4). In total, we observed deficiencies in either the ability to form wild-type-sized and/or mixed strain aggregates in all 12 of the transposon mutants we tested. Together, these observations are consistent with our hypothesis that the genes identified in our transposon screen are required for wild-type-like aggregation, and therefore necessary for T6SS2 function in hydrogel.

### Motility is necessary but not sufficient for aggregate formation

Given that the two flagellar motility mutants were unable to form substantial aggregates (Fig. 2D), we predicted that motility was required for aggregate formation in hydrogel. To test this prediction, we performed motility assays in LBS supplemented with 0.3% agar to quantify motility of each strain at the population level. As expected, the two flagellar mutants had significantly reduced motility relative to the wild type, and the *flgK* mutant did not display any motility during the experiment (Fig. 2D). The majority of conditional mutant strains tested also had significantly reduced motility compared with the wild type (Fig. 2D). However, three surface modification mutants had wild-type levels of motility yet made significantly smaller aggregates, suggesting that motility is required, yet not sufficient, to facilitate wild-type aggregation in hydrogel.

### Scanning electron microscopy (SEM) confirms lack of flagella and aggregation for the *flgK* mutant

**Fig 3:**
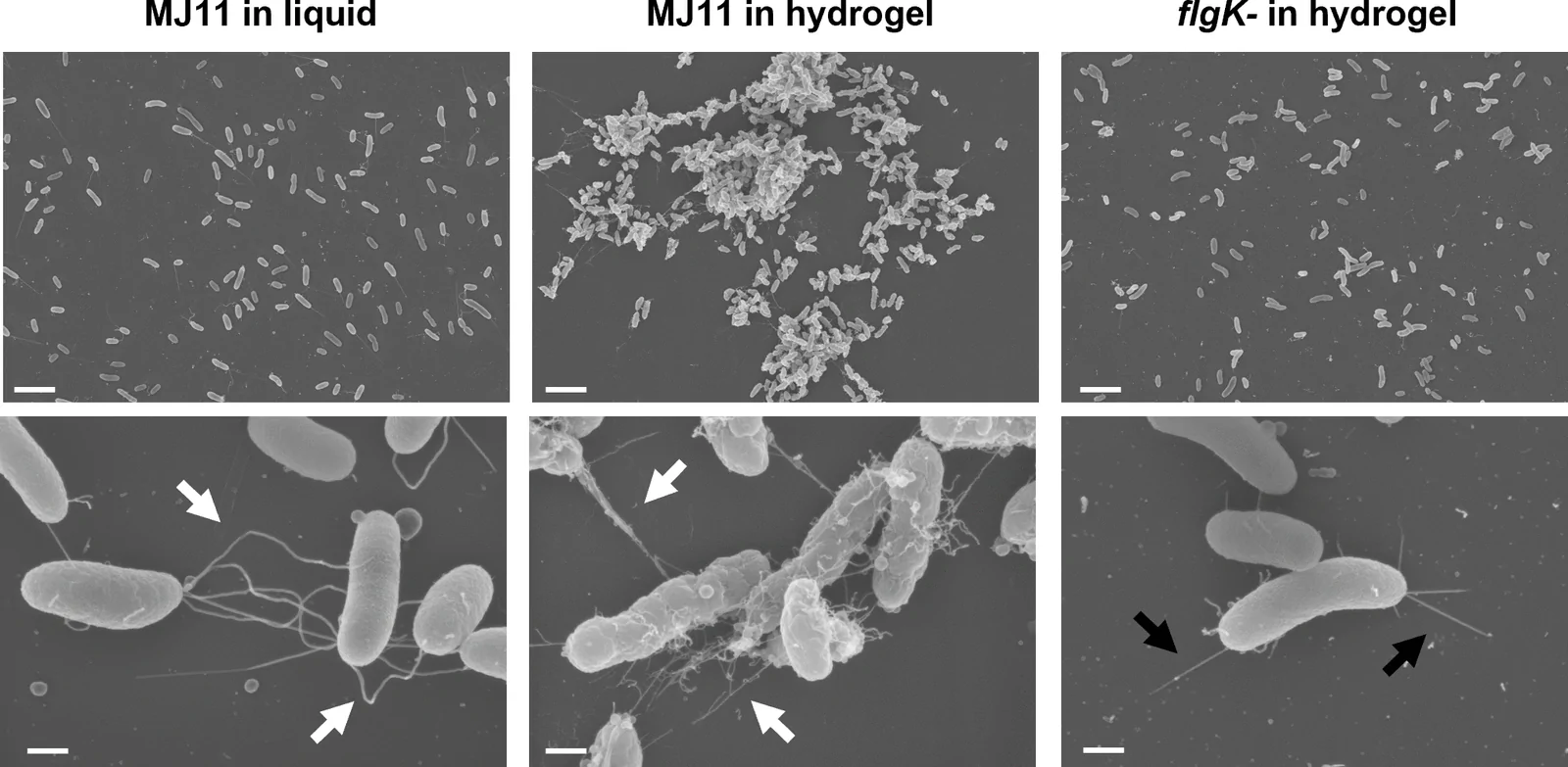
SEM images of WT and *flgK* mutants**.** Representative SEM images of indicated strains grown in liquid LBS or hydrogel (LBS + 5% PVP). White arrows indicate flagella. Black arrows indicate other extracellular structures resembling pilus-like structures. Scale bars are 4 µm (top row) and 400 nm (bottom row).

To directly observe aggregation and the impact of the *flgK* mutation, we subjected wild-type cells grown in liquid and hydrogel, as well as the *flgK* mutant grown in hydrogel, to SEM. This approach confirmed that only WT cells grown in hydrogel made aggregates (Fig. 3, top row). At higher magnification, WT flagella were observed in both liquid and hydrogel (Fig. 3, white arrows), while the *flgK* mutant did not produce flagella, although pilus-like structures were still visible (Fig. 3, black arrows). Furthermore, comparison of the high magnification images for the WT and *flgK* mutant grown in hydrogel revealed a difference in cell surface morphology. WT cells had a more textured surface, while the *flgK* mutant surface was smooth, similar to what was observed for the WT cells in liquid. These findings suggest that the molecular landscape of the cell surface may be influenced by both environmental conditions and genotype.

### Flagella and surface modification genes are required for TasL-dependent aggregation and killing in hydrogel

We next sought to understand how these new genetic determinants of aggregation might influence TasL*-*dependent cell contact. Given that TasL mediates aggregation in a heterotypic manner, we hypothesized that the *flgK* and surface modification mutations may prevent aggregation (and therefore killing) because the mutations may impact the ability of TasL to promote cell-cell contact between inhibitor and target cells in hydrogel. To test whether TasL is still functional in select mutants, we employed a series of coculture experiments to assess the ability of mixed cultures to form large, mixed-strain aggregates and restore killing in hydrogel. We reasoned that if mutations in *flgK* or a surface modification gene (specifically VFMJ11_0174) impact TasL function, then coincubation of these mutants with a *tasL* mutant would not restore aggregation or target cell killing. However, if the cocultures with *tasL−* cells result in large aggregates through extracellular complementation, this result would suggest that the *flgK* or 0174 mutant retains a functional TasL and either the TasL-binding partner, or other aggregation factor, is impacted. We chose LAS14H5, which has a transposon insertion in VFMJ11_0174, because it was impaired in aggregation abilities but still motile (Fig. 2), suggesting it has functional flagella.

**Fig 4:**
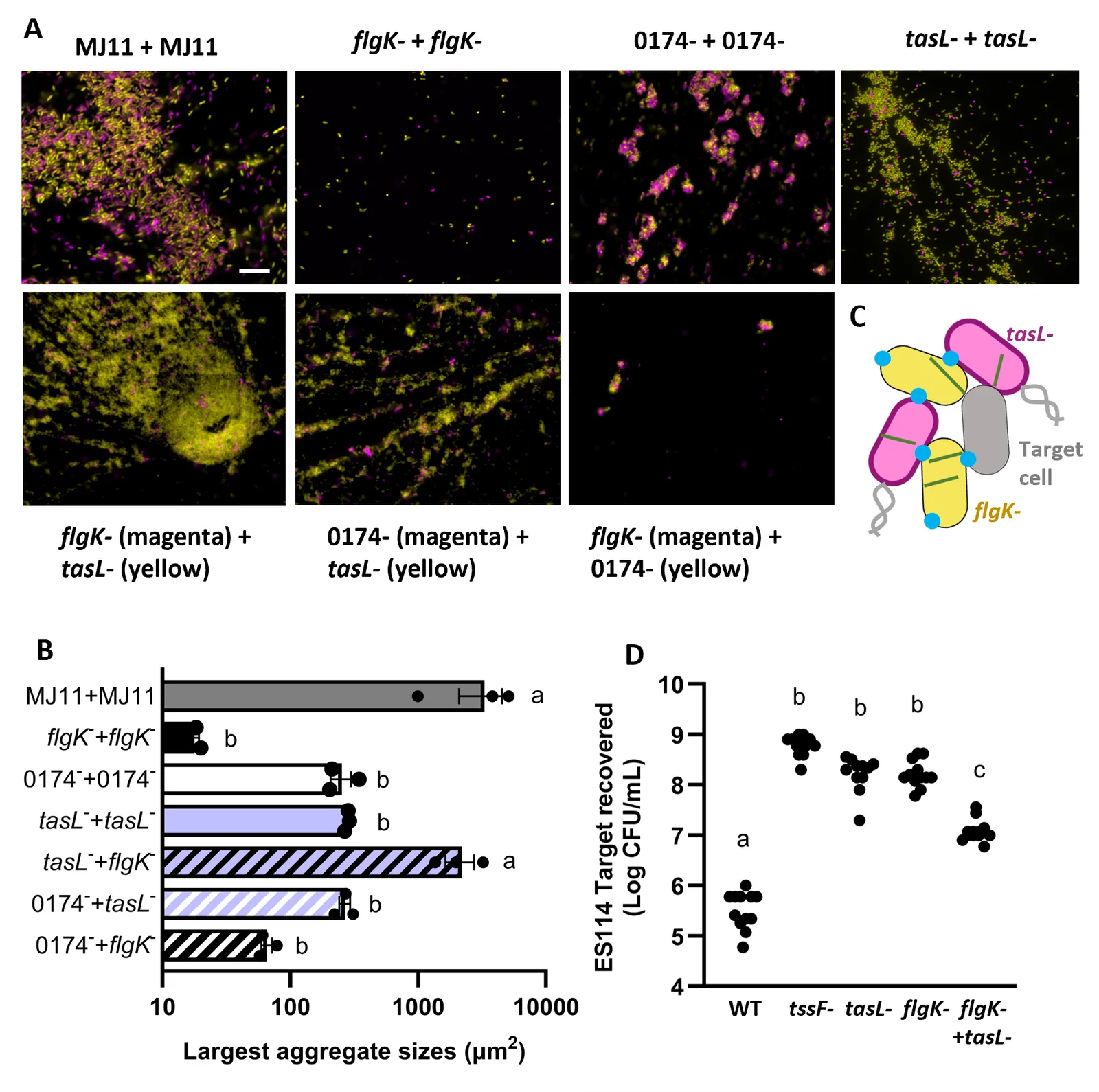
Flagella and surface modification genes are required for TasL-dependent aggregation and killing in hydrogel. (**A**) Representative images of the largest aggregates observed in coincubations of indicated differentially tagged strains in hydrogel. Scale bar is 10 µm. (**B**) Estimated aggregate size for the largest observed aggregates. Letters indicate significantly different sizes (one-way analysis of variance (ANOVA) with a Dunnett’s multiple comparison test; *P* < 0.01. (**C**) Model for how coculture of *tasL* and *flgK* mutants can complement aggregation and target killing in hydrogel. (**D**) Results from coincubation assays between ES114 (target) and MJ11 reported as the amount of target CFUs recovered after 24 h (log CFU/mL). MJ11 genotype is shown on the *x*-axis: wild-type (WT), *tssF* mutant (*tssF*−), *tasL* mutant (*tasL*−), and *tasL* and *flgK* mutants simultaneously (*tasL− flgK*−). Letters indicate significantly different target recovery values between treatments (one-way analysis of variance [ANOVA] with Tukey’s multiple comparison test; *P* < 0.0001). Each experiment was performed four times with three biological reps (*n* = 12).

Experimental results suggest that the 0174 mutant has impaired TasL function; however, the *flgK* mutant (partially) rescued the *tasL*− phenotype. When clonal, differentially-tagged *tasL*, 0174, or *flgK*- mutants are coincubated in hydrogel, aggregates were significantly smaller than the wild-type parent (Fig. 4A and B). However, if the *flgK* mutant is mixed with the *tasL* mutant, large aggregates are observed (Fig. 4A) that were not significantly different in size compared with the wild-type parent aggregates (Fig. 4B). Moreover, while the *tssF*, *tasL*, or *flgK* mutants cannot kill ES114 target in hydrogel, coincubating target cells with a mix of *flgK* and *tasL* mutants partially restores killing ability (Fig. 4D), likely due to restored aggregation (Fig. 4C). These results suggest that the *flgK* mutant is not deficient at aggregation due to lack of TasL function.

Coincubation assays with the 0174 mutant suggest it may be impaired in both heterotypic components of TasL aggregation. When the 0174 mutant was coincubated with *tasL*− cells, large aggregates were not observed (Fig. 4B), suggesting TasL function is impaired in the 0174 mutant. Moreover, large aggregates were still not observed in coincubations with the 0174 mutant and the *flgK* mutant (Fig. 4B), which appears to have a functional TasL, suggesting the 0174 mutant may also lack an exposed ligand for TasL binding.

Taken together, these findings indicate that the new genetic determinants identified in this screen, flagella and the surface modification gene cluster (specifically VFMJ11_0174), are needed for the heterotypic interactions with TasL during aggregation (Fig. 4). Based on these findings, one possible model is that flagella may be required (directly or indirectly) for cells to sense changes in the environment (i.e. increased viscosity) to make modifications to the cell surface that engage in TasL-dependent aggregation. Therefore, to better understand how flagella are connected to aggregation in hydrogel, we sequenced the transcriptomes of WT and *flgK* mutants grown in liquid and hydrogel.

### *flgK* is required for transcriptional up-shift during habitat transition

Given that flagella play a key role in environmental sensing in other bacteria (20, 21) and are required in *V. fischeri* for aggregation in hydrogel (Fig. 2 and 3), we sought to investigate the regulatory role of flagella during habitat transition. Genome resequencing determined no secondary mutations in genes related to aggregation factors (Fig. S5). Moreover, when the *flgK* transposon mutation was moved into a fresh MJ11 background using natural transformation (22), the remade *flgK* mutant (MP101) showed similar aggregation deficiencies compared with the original *flgK* mutant (LAS22A10) (Fig. S6), indicating that *flgK* is required for proper aggregation.

**Fig 5:**
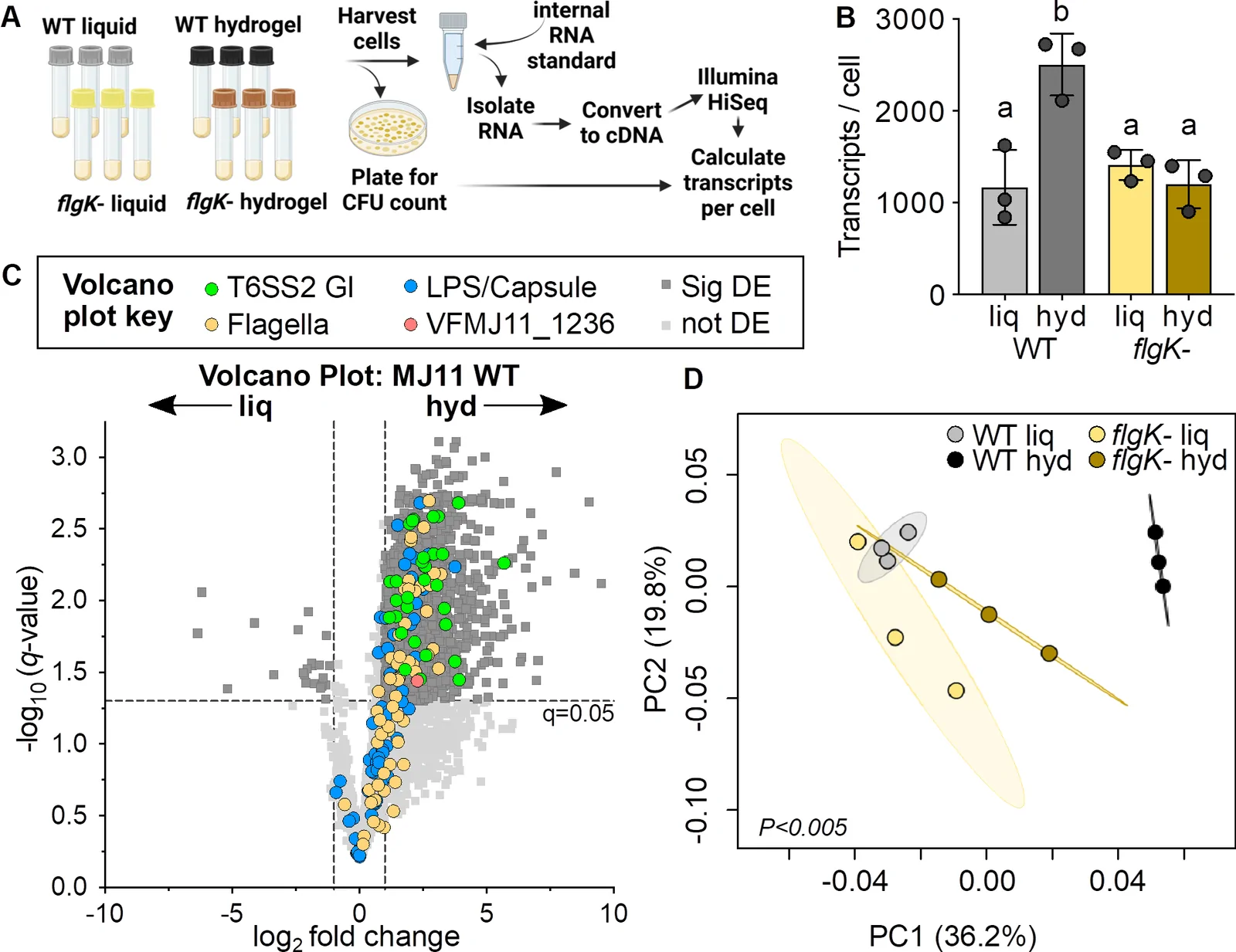
MJ11 gene expression increases dramatically during transition from liquid to hydrogel in a flagellum-dependent manner. (**A**) Schematic of experimental design to produce transcriptomes for MJ11 wild-type and *flgK* mutant (*flgK*−) in liquid or hydrogel (made with Biorender.com). (**B**) Number of transcripts per cell for MJ11 transcriptomes for the wild-type (WT) or *flgK* mutant (*flgK*−) incubated in liquid LBS (liq) or hydrogel (hyd). Each symbol represents an individual sample (transcriptome); letters indicate significantly different values of transcripts per cell between sample groups (two-way analysis of variance [ANOVA] with Šídák’s multiple comparison post-test, *P* < 0.02). (**C**) Volcano plot showing log_2_ fold difference in transcript abundance between liquid and hydrogel for MJ11 wild-type transcriptomes. Each point represents expression of a single gene. Genes with a negative log_2_ fold change have higher expression in liquid (left), while genes with a positive log_2_ fold change have higher expression in hydrogel (right). Colors indicate the functional assignment of genes of interest: green, T6SS2 genomic island (GI); yellow, flagella; blue, LPS/capsule; red, VFMJ11_1236. Data points above the dashed horizontal line had significant *q*-values between conditions (Student’s *t*-test with correction for false discovery rate, *q* < 0.05), and those outside the vertical dashed lines had a magnitude fold change of >|1| log_2_ between conditions (dark gray squares). (**D**) Principal coordinates analysis (PCoA) plot of transcriptome data. The percentages on each axis indicate the amount of variation explained by the axis and ellipses indicate 95% confidence intervals. Adonis *P* value for comparison between transcriptomes is shown in the lower left-hand corner.

We employed quantitative transcriptomics to achieve two specific aims: (i) determine how habitat transition impacts *V. fischeri* transcript abundance at the per cell level, and (ii) describe the role of flagella in mediating the observed changes in gene expression. We modified methods described from Gifford et al*.* (23, 24) to produce quantitative transcriptomes for *V. fischeri* MJ11 wild-type and a flagellar mutant (*flgK*−) identified in our screen (Fig. 5A). Briefly, RNA was collected from monocultures of each strain grown in liquid or hydrogel for 12 h, when cells are still in exponential growth (12). Quantitative transcriptomics was achieved by addition of internal RNA standards just prior to RNA extraction (see Materials and Methods and Text S1 for an in-depth explanation). Internal standard transcript sequences were recovered and used to convert the number of reads per library to number of transcripts per cell, based on CFU values. Moreover, comparing these CFU counts for each treatment revealed no significant difference, suggesting that comparable growth and number of cells were used to harvest RNA (Fig. S7A).

To obtain a high-level understanding of how cells transcriptionally respond to the physical environment, we first asked whether strain genotype and/or medium viscosity affected the amount of mRNA per cell. We evaluated the number of transcripts per cell for each strain in each condition, and observed that on average, wild-type liquid samples had 1,165 transcripts per cell (Fig. 5B ; Fig. S7B), which is comparable to other bacterial species during exponential growth in a lab setting (*E. coli:* 1,380–1,800 mRNAs/cell) (25, 26). Unexpectedly, wild-type hydrogel samples had 2,501 transcripts per cell, more than twice as many as in liquid (Fig. 5B; Fig. S7B). The *flgK* mutant had 1,410 and 1,196 transcripts per cell in liquid and hydrogel, respectively (Fig. 5B). The *flgK* mutant mRNA abundance values were not significantly different from the wild type in liquid but were significantly lower than the wild type in hydrogel (Fig. 5B). Thus, the *flgK* mutation prevented an increase in transcription in hydrogel grown cells. We next performed a principal coordinates analysis (PCoA) on the MJ11 transcriptomes and found that *flgK* mutant samples clustered with wild-type liquid samples and separately from wild-type hydrogel samples (Fig. 5D; ANOVA: *P* < 0.005). Taken together, these data suggest that flagella contribute significantly to MJ11’s transcriptional response to increased environmental viscosity.

To identify which transcripts were driving this change in gene expression for wild-type cells, we generated a volcano plot comparing transcript abundance for each gene between liquid and hydrogel (Fig. 5C). Approximately 59% of the genome (2,451 genes) was significantly differentially expressed between culture conditions, and most of those genes (2,429 or 99% of differentially expressed genes) had higher expression in hydrogel, while only 22 genes (1% of differentially expressed genes) had higher expression in liquid (Fig. 5C). This observation suggests that MJ11 upregulates over half of its genome based on a mechanical signal (viscosity) that changes as cells transition from free-living to a host-associated lifestyle. Notable gene transcripts that were significantly more abundant in hydrogel included: (i) T6SS2 genes, which we expected based on previous work (12); (ii) surface modification and flagellar synthesis gene clusters, which we obtained transposon insertions in for this screen (Fig. 5C); (iii) 94 predicted transcriptional regulators; and (iv) host-relevant metabolic and detoxification pathways. Therefore, we next used hierarchical clustering analysis to determine whether differences in gene expression are driven by condition (liquid vs hydrogel), genotype (WT vs *flgK*−), or a combination of both.

### Flagella promote expression of surface modification genes in hydrogel

**Fig 6:**
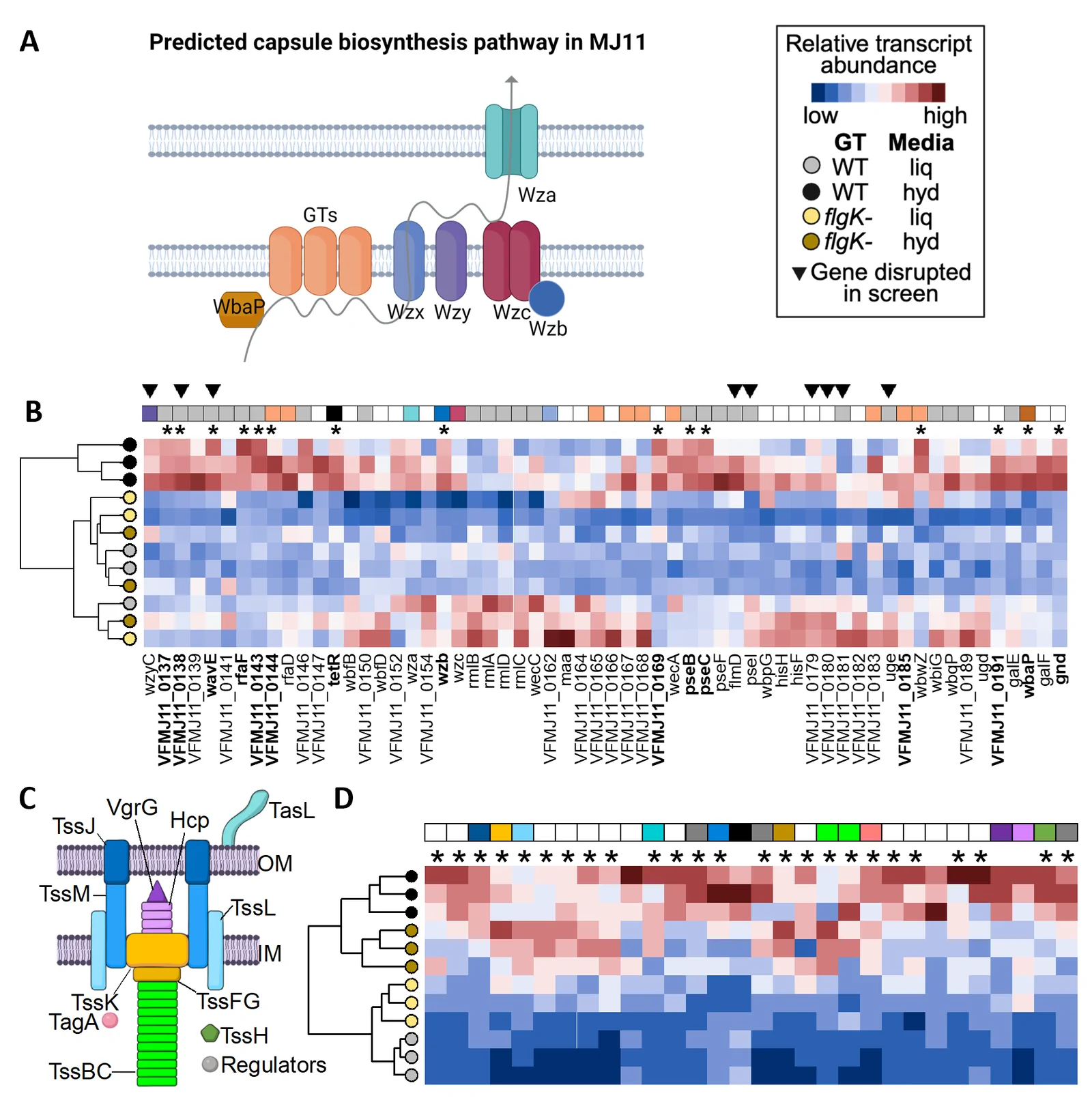
Flagellum-dependent gene expression for competition factors in liquid or hydrogel. Hierarchical clustering analysis and heatmap displaying transcript abundance for each gene for the surface modification (**A and B**) and T6SS2 (**C and D**) gene clusters. Circle color indicates the strain genotype and medium condition: light gray (wild-type [WT] in liquid [liq]), dark gray (wild-type in hydrogel [hyd]), light yellow (*flgK* mutant [*flgK*−] in liquid), and dark yellow (*flgK* mutant in hydrogel). Transcript abundance is shown as relative transcript abundance where values are scaled across each gene (column); blue indicates relatively low transcript abundance while red indicates relatively high abundance. For panels B and D, squares above each column (gene) indicate its known or predicted function based on associated schematic (**A and C**); OM, outer membrane; IM, inner membrane. Gray boxes indicate predicted polysaccharide synthesis genes. Black boxes indicate predicted transcriptional regulators. For panel **B**, asterisks indicate expression of a gene was significantly higher for wild-type hydrogel samples compared with all other samples (one-way ANOVA, *P* < 0.03). For panel **D**, asterisks indicate expression of a gene was significantly higher for hydrogel samples compared with liquid samples (Student’s *t*-test, *P* < 0.01). Gene ID (VFMJ11_#) for each heatmap are shown left to right as follows: A0804–A0833. Figure made with Biorender.com

Our transcriptomes revealed genes in the surface modification cluster identified in our transposon screen are also significantly upregulated in the wild type in hydrogel. This genomic region, for which we isolated transposon mutants with impaired aggregation and killing, encodes gene products predicted to be involved in LPS modification, synthesis of predicted surface-exposed glycans such as pseudaminic acid, and homologs of components of the Wxz/Wxy capsule biosynthesis and export system (Fig. 6A) (27). Hierarchical cluster analysis of transcript abundance for genes in this genomic region revealed that wild-type hydrogel samples clustered separately from all other samples, while wild-type liquid, *flgK* mutant liquid, and *flgK* mutant hydrogel samples clustered together (Fig. 6B). On average, wild-type hydrogel samples had higher gene expression compared with the other samples, although this was only statistically significant for 15 of 60 genes (Fig. 6B, asterisks). Taken together, these findings support three main conclusions: (i) surface modification genes are generally upregulated in wild-type upon transition from low to high viscosity liquid, (ii) this increase in gene expression is dependent on *flgK*, and (iii) these surface modification genes are necessary for aggregation required for T6SS-dependent killing in hydrogel. These results are consistent with our findings in Fig. 3 that revealed distinct cell surface morphologies depending on the genotype and growth condition.

A hierarchical cluster analysis of the T6SS2 gene cluster grouped samples by medium condition rather than strain genotype (Fig. 6C and D). This observation indicates that strain genotype did not influence sample clustering, which is consistent with our above findings that the flagellar components *flgK* and *flgB* are not required for T6SS2 sheath assembly or function on agar surfaces. Together, these data indicate that flagella are not required for T6SS2 activation in hydrogel or on surfaces, suggesting that the T6SS weapon is activated by a different sensory mechanism that is coordinated with flagellum-dependent expression of predicted surface modification genes. Indeed, pili were still observed in the *flgK* SEM images (Fig. 3), suggesting that additional mechanosensory mechanisms require exploration. Because *V. fischeri* genomes encode multiple pilus structures, it is possible that our screen would not have identified these, or other mechanical sensors that may have functional redundancy.

### Flagella promote transcriptional changes of symbiotically relevant genes

Given our previous observations that environmental viscosity primes *V. fischeri* cells for T6SS killing within host-like environments (12), we predicted that the viscosity signal may prime cells for other host-relevant functions. Therefore, we searched the transcriptomes for genes with previously characterized, host-relevant functions that showed *flgK*-dependent increased transcription in hydrogel relative to liquid media. This analysis identified genes relating to motility, chemotaxis, carbon and iron transport, and nitric oxide detoxification (Fig. S8).

## DISCUSSION

This work combines a genome-wide screen with transcriptomics to show the key role of flagella in facilitating competitive interactions in a liquid environment. Through quantitative transcriptomics, we determined that *V. fischeri* dramatically alters its cellular mRNA content by elevating global transcription during simulated habitat transition between free-living and host-associated lifestyles. Our evidence suggests that flagella are required for *V. fischeri* to appropriately respond to changes in environmental viscosity during habitat transition in two important ways: (i) promoting expression of surface modification genes required for aggregation necessary for T6SS-mediated killing and (ii) modulating expression of other symbiotically relevant genes.

Motility and chemotaxis are required for host colonization in *V. fischeri* (28–31). We observed that 18 out of 54 flagella genes had significantly higher transcript abundance in wild-type hydrogel samples relative to wild-type liquid and *flgK* mutant samples (Fig. S8A). These data are consistent with previously published proteomes where flagellum-related proteins, such as filaments, were more abundant in cells grown in hydrogel (12), and suggest flagella may be required to enhance their own expression during habitat transition. We also observed a *flgK*-dependent increase in transcript abundance in hydrogel for chemotaxis-related genes including methyl-accepting chemotaxis proteins and corresponding regulatory proteins CheR and CheW (Fig. S8; Table S1).

Previous work also uncovered some of the nutrients that are available or limiting within the host light organ, including the presence of mannose (32), N-acetylglucosamine (33), amino acids (34), and haem, which can serve as an iron source for *V. fischeri* (35). We found the transcripts for N-acetylglucosamine and mannose-specific PTS genes, as well as branched-chain amino acid transporter genes, were expressed up to 10-fold higher in wild-type hydrogel samples compared with other treatments (Fig. S8B). Furthermore, transcript abundance for synthesis of the aerobactin siderophore and transporter gene cluster (36, 37), as well as the heme uptake genes (35), was also highly expressed in hydrogel (Fig. S8B). Because cells appear to be expressing carbon and iron acquisition pathways under conditions where they are not directly experiencing iron limitation (LBS + PVP) or the presence of these specific sugars, these results suggest that higher viscosity may be a signal to prepare for the nutrient conditions that are encountered in the host.

Finally, *V. fischeri* cells encounter host-derived nitric oxide (NO) and require detoxification mechanisms to promote colonization (38–42). Interestingly, we found that hydrogel stimulates expression of two activators (*norR_1* and *norR_2*) of NO detoxification pathways in other organisms: cytochrome c-554 and nitric oxide reductase (*hcp/hcr*) (43–45) (Fig. S8B). However, expression of *V. fischeri* genes that are known to be induced by NO (e.g., *aox*) were not elevated in our transcriptomes (42, 46) (Table S1), indicating that the cells are not experiencing NO stress but rather preparing for it, in response to transition to high viscosity. Taken together, these transcriptional effects suggest that flagella are necessary to coordinately activate expression of key competition and colonization factors in *V. fischeri*.

Our findings also provide a possible explanation for why T6SS2 in *V. fischeri* requires the Sigma 54-dependent transcriptional regulator VasH to fully activate T6SS2. Previous work showed the T6SS2-encoding *V. fischeri* strain FQ-A001 requires *rpoN*, which encodes the Sigma-54 cofactor, for both T6SS2 function and motility (47). Our data, which use a different *V. fischeri* strain, provide a possible ecological explanation for why T6SS2 function and motility may be coordinately regulated via Sigma-54: to facilitate cell surface modifications that promote aggregation, which is required for T6SS-mediated killing in liquid environments. Similar mechanisms of coordinate regulation have been observed in other taxa where sigma factors play important roles in coregulating virulence factors such as T6SS and flagellar motility (48–51).

Although the mechanism by which flagella activate transcriptional and functional changes during habitat transition for *V. fischeri* remains unknown, our findings are consistent with flagella playing some role in sensing changes in a mechanical signal in its environment. Direct and indirect evidence for flagella as a mechanosensor has been described for *E. coli*, *Proteus*, *Pseudomonas*, *Vibrio cholerae,* and *Vibrio parahaemolyticus* (52–54). For these model organisms, the flagella are needed to sense the physical environment and initiate highly regulated, sequential processes that direct coordinated cellular behaviors such as biofilm and swarming motility. In the case of *V. parahaemolyticus*, the closest relative of *V. fischeri* in this group, mechanical inhibition of the flagella rotation due to high viscosities, surfaces, or functional disruption (via phenamil or mutation), can trigger swarmer cell differentiation (55, 56), elicit upregulation of colonization and virulence factors (57), as well as increased expression and secretion of the T6SS protein Hcp1 (58). Similarly, our data support a model whereby *V. fischeri* flagella are required to elicit a transcriptional response to host-like viscosity to coordinate multiple behaviors that are required for successful competitive colonization of a host. Future work may identify the mechanism(s) by which *V. fischeri* detects changes in environmental viscosity, as well as additional sensors that activate the T6SS weapon.

**Fig 7:**
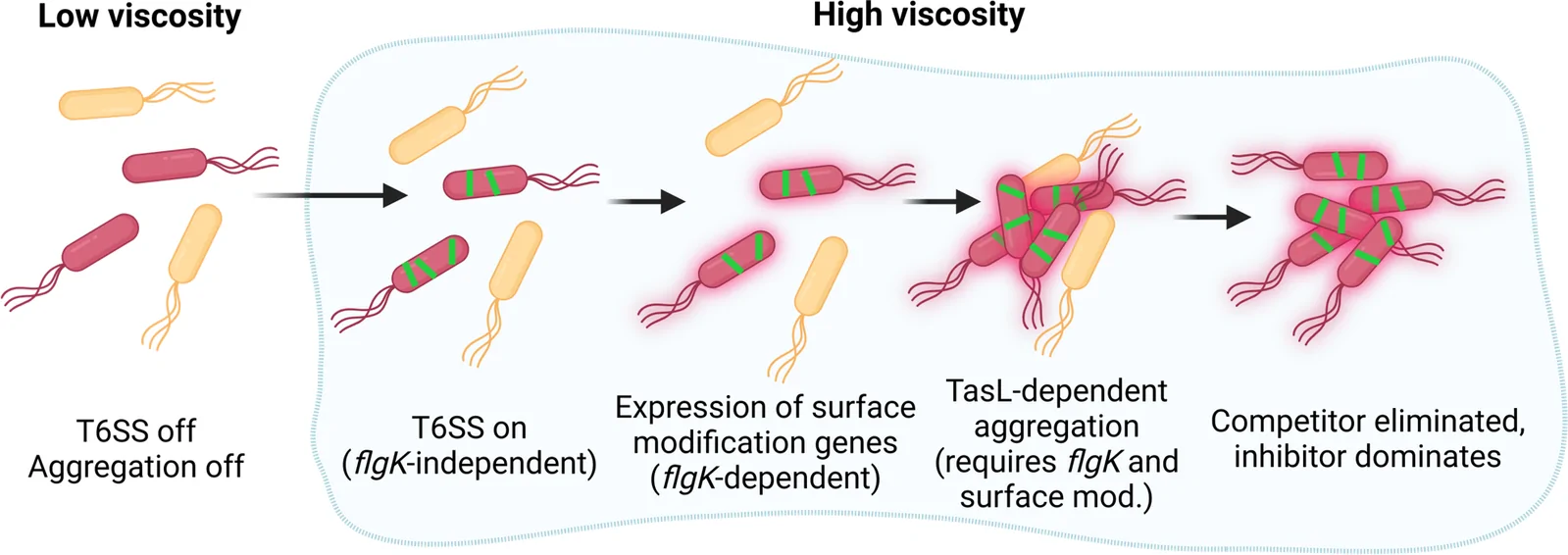
Model for how flagella influence cell-cell contact for T6SS-competition in high viscosity liquid. Transition from low viscosity (LBS liquid) to high viscosity (LBS PVP) activates T6SS2 (*flgK*-independent) as well as surface modifications (*flgK* dependent) for TasL-mediated aggregation that facilitates T6SS-mediated killing of competitor strain. Figure made with Biorender.com.

In conclusion, this work identifies factors that promote contact-dependent competition in a viscous liquid and reveals how the physical environment influences expression of symbiotically relevant genes. Based on these and previous findings, we propose an updated model for how *V. fischeri* cells mediate cell-cell contact to engage in T6SS-mediated competition during habitat transition (Fig. 7). As cells transition from low to high viscosity conditions, the T6SS2 is activated, and flagella are required for enhanced expression of cell surface modification genes required for mediating TasL-dependent aggregation. The cell contact between T6SS+ inhibitor and competitor cells allows inhibitors to eliminate the competitor and dominate the habitat.

## MATERIALS AND METHODS

See Text S1 for additional methods and experimental details, specifically medium and growth conditions, strains and plasmids, details about the screening transposon mutants, coincubation assays, motility assays, and fluorescence microscopy.

### Transposon mutagenesis and screen for conditional mutants

Conjugations were set up between *V. fischeri* strain MJ11 (15) and the mini-Tn5-ermR transposon vector pEVS170, which was maintained in *E. coli* strain RHO3, a DAP auxotroph (59). After a 24-h incubation period on Luria Bertani with added salt (LBS) agar supplemented with DAP at 24°C, mating spots were resuspended in 100 μL LBS medium and spread plated onto LBS agar medium containing erythromycin (erm) (see supplemental methods for more details). A total of 7,300 colonies were picked and screened for the ability to kill *V. fischeri* strain ES114 in hydrogel (LBS broth with 5% [w/vol] PVP) yet not on LBS agar plates.

High throughput coincubation assays in hydrogel were performed for each mutant and a GFP-tagged target strain, as described previously (11). Strains were mixed in a 9:1 (MJ11 tn5 mutant:ES114 pVSV102) ratio and incubated standing in LBS hydrogel for 24 h in a 96-well plate at 24°C. Luminescence, fluorescence, and absorbance values collected by a Tecan Plate reader were used to assess the killing ability of each mutant. Only mutants from wells with bright luminescence (indicating MJ11 grew) and GFP fluorescence (indicating the target grew) values comparable to those from the control containing ES114 pVSV102 and MJ11 *tssF_2* mutant strain LAS04 were selected for further study. Of the 7,300 mutants screened, 222 did not kill ES114 in hydrogel. We validated this phenotype by performing coincubation assays in hydrogel and quantifying CFUs of target strain (12), and moved forward with 45 mutants. We then performed agar plate coincubation assays, as described in references 5 and 60, between each mutant and GFP-tagged ES114 to determine whether each mutant prevented the growth of ES114 when contact was forced. Twenty of these mutants prevented the growth of ES114 on agar plates.

### Mapping transposon insertion sites

To identify where the transposon inserted in each mutant, we performed cloning, as described in reference 61, and inverse PCR (iPCR). For all transposon mutants, chromosomal DNA was digested with the HhaI restriction enzyme, fragments were self-ligated with T4 DNA ligase. For cloning, the resulting circularized DNA was transformed into *E. coli* DH5⍺λpir and plated onto BHI Erm to select for transformants containing the Erm resistance gene within the transposon. Plasmid minipreps were performed to purify the circularized transposon and flanking DNA, and Sanger sequencing was used to sequence across the transposon-chromosome junction using primer M13F. For iPCR, the circularized gDNA from T4 DNA ligase reactions was used as the template for PCR amplification with primers specific for the transposon: AS1100 (TACATTGAGCAACTGACTGAAATGCC) and EXT170 (GCACTGAGAAGCCCTTAGAGCC). These primers sequence outward from the transposon, around the piece of circularized DNA, such that the resulting PCR product is a combination of the transposon and the disrupted gene. iPCR products were cleaned with a Zymo Clean/Concentrate kit followed by Sanger sequencing.

### Gene cluster analysis

The surface modification gene cluster (*mutM* to *galE*) was extracted from indicated *V. fischeri* genomes using Geneious and compared using CAGEGATE (62), and clinker (63) was used to annotate the visual output. All sequences are publicly available through GenBank except for MJ1, which we obtained from long reads from Plasmidsaurus and provide as Text S2.

### LAS22A10 genome resequencing

LAS22A10 gDNA was extracted using a Zymogen Bacterial and Fungi DNA extraction kit, quantified with a QuantIT kit, and sent for Illumina sequencing to SeqCenter LLC (Pittsburgh, PA). Using Geneious software, reads were trimmed, error corrected, duplicate reads removed, and mapped against the MJ11 reference genome. Mutations in the LAS22A10 reads were identified using the Single Nucleotide Polymorphism (SNPs) feature in Geneious, with default settings and 100% variant frequency. Mutation locations were confirmed using NCBI Blast.

### Natural transformation

To construct strain MP101, MJ11 pLostfox-Kan was prepared, as previously described (22, Text S1) and transformed with gDNA extracted from LAS22A10 (*flgK*:tn5). Transformants were screened for erm resistance and Kan sensitivity to ensure transfer of the transposon insertion and loss of the pLostfox-Kan plasmid.

### Transcriptome collection and data analysis

Overnight cultures of MJ11 were subcultured into fresh LBS liquid or hydrogel and grown for 12 h at 24°C. To dilute the PVP and stop transcription, 500 μL of each sample was added to a pre-chilled tube containing 3 mL LBS medium and 1 mL stop solution (5% [vol/vol] phenol, pH 4.3, and 95% [vol/vol] ethanol). Cells were pelleted through centrifugation (4k rpm for 15 min at 4°C), washed with 500 µL LBS, and pellets were frozen overnight at −80°C. CFUs were quantified for each sample (three technical replicates per sample) prior to pelleting for downstream calculations. RNA was then extracted using the MirVana kit following the manufacturer’s protocol (Thermo Fisher Scientific, Waltham, MA). *Saccharolobus solfataricus* P2 RNAs were used as internal RNA standards (IS), which were added after adding lysis buffer and prior to adding the miRNA homogenate additive; see Text S1 for IS details. Residual DNA was removed via the Turbo DNA-free kit (Invitrogen, Carlsbad, CA). cDNA library prep and sequencing were performed at the University of North Carolina (UNC) High-Throughput Sequencing Facility (HTSF) with the HiSeq 4000 platform (single-end 50-bp reads). Three biological replicates for each strain genotype and treatment were sequenced (*n* = 12). Quality scores were calculated using FastQC; low-quality sequences (average quality score < 20 across 5 bp) were removed using Trimmomatic (64). rRNA and IS RNA were counted and removed from the data set with BLAST. Reads were mapped to the MJ11 genome using BowTie2 (65) and counted using HTSeq (66). Methods described in references 23 and 24 were used to quantify MJ11 mRNA within each sample. A recovery factor for each IS within a sample was calculated by taking the amount of individual IS added and dividing by the number of IS transcripts recovered. Recovery factors for the IS were averaged for each sample. Read counts for each gene were then multiplied by the average recovery factor for each sample and then divided by the average number of CFUs (cells) for each sample to calculate the total number of transcripts per cell for each sample (see Table S1 for calculations).

Differential expression analysis was performed, as described previously (12, 67). This involved a contingency table that was generated by comparing average transcript per CFU abundance between treatments using a Student’s *t*-test corrected for multiple comparisons using the false discovery rate (FDR). Volcano plots were generated by graphing the negative log_10_ *q*-value and log_2_ fold change between treatments. Because zero values cannot be log-transformed for differential expression analysis, zero values were set to the limit of detection for a given gene, which was one read per gene. Only transcripts with a log_10_*q*-value ≤0.05 and a log_2_ fold change ≥|1| are considered statistically significantly differentially expressed (DE). Hierarchical clustering analysis was performed using the heatmap function in R; transcript per 1,000 cell values were not scaled. The vegan package in R was used to perform principal coordinates analysis (PCoA) using the Bray-Curtis dissimilarity index, PerMANOVA using the Adonis function (68). Dispersion was determined using the “permutest.betadisper” function to ensure there were no significant differences in dispersion between the groups being compared.

## Data Availability

Transcriptome sequences were submitted under Bioproject number PRJNA1013100.

## SUPPLEMENTAL MATERIAL

Fig. S1 mbio.03970-24-s0001.tif Surface modification genes show significant strain variation.

Fig. S2 mbio.03970-24-s0002.tif Conditional mutants can assemble T6SS2 sheaths.

Fig. S3 mbio.03970-24-s0003.tif Conditional mutants show reduced aggregation in hydrogel.

Fig. S4 mbio.03970-24-s0004.tif The majority of conditional mutants form mixed aggregates.

Fig. S5 mbio.03970-24-s0005.tif LAS22A10 sequencing results.

Fig. S6 mbio.03970-24-s0006.tif Aggregation phenotypes for original and remade mutants.

Fig. S7 mbio.03970-24-s0007.tif Statistics for transcriptomes.

Fig. S8 mbio.03970-24-s0008.tif Flagella promote gene expression changes in hydrogel.

Supplemental Methods mbio.03970-24-s0009.docx Texts S1 and S2.

Table S1 mbio.03970-24-s0010.xlsx Transcript abundance data.

ASM does not own the copyrights to Supplemental Material that may be linked to, or accessed through, an article. The authors have granted ASM a non-exclusive, world-wide license to publish the Supplemental Material files. Please contact the corresponding author directly for reuse.

## References

[1] Granato ET, Meiller-Legrand TA, Foster KR. 2019. The evolution and ecology of bacterial warfare. Curr Biol 29:R521–R537. doi:10.1016/j.cub.2019.04.024 31163166

[2] Klein TA, Ahmad S, Whitney JC. 2020. Contact-dependent interbacterial antagonism mediated by protein secretion machines. Trends Microbiol 28:387–400. doi:10.1016/j.tim.2020.01.003 32298616

[3] Boyer F, Fichant G, Berthod J, Vandenbrouck Y, Attree I. 2009. Dissecting the bacterial type VI secretion system by a genome wide in silico analysis: what can be learned from available microbial genomic resources? BMC Genomics 10:1–14. doi:10.1186/1471-2164-10-104 PMC2660368 19284603

[4] Suria AM, Smith S, Speare L, Chen Y, Chien I, Clark EG, Krueger M, Warwick AM, Wilkins H, Septer AN. 2022. Prevalence and diversity of type VI secretion systems in a model beneficial symbiosis. Front Microbiol 13:988044. doi:10.3389/fmicb.2022.988044 PMC9515649 36187973

[5] Speare L, Cecere AG, Guckes KR, Smith S, Wollenberg MS, Mandel MJ, Miyashiro T, Septer AN. 2018. Bacterial symbionts use a type VI secretion system to eliminate competitors in their natural host. Proc Natl Acad Sci USA 115:E8528–E8537. doi:10.1073/pnas.1808302115 PMC6130350 30127013

[6] Logan SL, Thomas J, Yan J, Baker RP, Shields DS, Xavier JB, Hammer BK, Parthasarathy R. 2018. The *Vibrio cholerae* type VI secretion system can modulate host intestinal mechanics to displace gut bacterial symbionts. Proc Natl Acad Sci USA 115:E3779–E3787. doi:10.1073/pnas.1720133115 PMC5910850 29610339

[7] García-Bayona L, Comstock LE. 2018. Bacterial antagonism in host-associated microbial communities. Science 361:eaat2456. doi:10.1126/science.aat2456 30237322

[8] Fu Y, Ho BT, Mekalanos JJ. 2018. Tracking *Vibrio cholerae* cell-cell interactions during infection reveals bacterial population dynamics within intestinal microenvironments. Cell Host Microbe 23:274–281. doi:10.1016/j.chom.2017.12.006 PMC6031135 29398650

[9] Hubert CL, Michell SL. 2020. A universal oyster infection model demonstrates that *Vibrio vulnificus* Type 6 secretion systems have antibacterial activity *in vivo*. Environ Microbiol 22:4381–4393. doi:10.1111/1462-2920.15123 32515159

[10] Cohen H, Fridman CM, Gerlic M, Salomon D. 2023. A *Vibrio* T6SS-mediated lethality in an aquatic animal model. Microbiol Spectr 11:e0109323. doi:10.1128/spectrum.01093-23 PMC10433976 37310285

[11] Speare L., Woo M, Dunn AK, Septer AN. 2022. A putative lipoprotein mediates cell-cell contact for type VI secretion system-dependent killing of specific competitors. mBio 13:e0308521. doi:10.1128/mbio.03085-21 PMC9040878 35404117

[12] Speare L., Smith S, Salvato F, Kleiner M, Septer AN. 2020. Environmental viscosity modulates interbacterial killing during habitat transition. mBio 11:e03060-19. doi:10.1128/mBio.03060-19 PMC7002345 32019799

[13] Speare Lauren, Woo M, Bultman KM, Mandel MJ, Wollenberg MS, Septer AN. 2021. Host-like conditions are required for t6ss-mediated competition among vibrio fischeri light organ symbionts. mSphere 6:e0128820. doi:10.1128/mSphere.01288-20 PMC8386388 34287008

[14] Speare L., Jackson A, Septer AN. 2022. Calcium promotes T6SS-mediated killing and aggregation between competing symbionts. Microbiol Spectr 10:e0139722. doi:10.1128/spectrum.01397-22 PMC9769598 36453912

[15] Mandel MJ, Wollenberg MS, Stabb EV, Visick KL, Ruby EG. 2009. A single regulatory gene is sufficient to alter bacterial host range. Nature 458:215–218. doi:10.1038/nature07660 PMC2713604 19182778

[16] Bose JL, Wollenberg MS, Colton DM, Mandel MJ, Septer AN, Dunn AK, Stabb EV. 2011. Contribution of rapid evolution of the *luxR-luxI* intergenic region to the diverse bioluminescence outputs of *Vibrio fischeri* strains isolated from different environments. Appl Environ Microbiol 77:2445–2457. doi:10.1128/AEM.02643-10 PMC3067414 21317265

[17] Phillips NJ, Adin DM, Stabb EV, McFall-Ngai MJ, Apicella MA, Gibson BW. 2011. The lipid A from *Vibrio fischeri lipopolysaccharide*: a unique structure bearing a phosphoglycerol moiety. J Biol Chem 286:21203–21219. doi:10.1074/jbc.M111.239475 PMC3122182 21498521

[18] Post DMB, Yu L, Krasity BC, Choudhury B, Mandel MJ, Brennan CA, Ruby EG, McFall-Ngai MJ, Gibson BW, Apicella MA. 2012. O-antigen and core carbohydrate of *Vibrio fischeri lipopolysaccharide:* composition and analysis of their role in *Euprymna scolopes* light organ colonization. J Biol Chem 287:8515–8530. doi:10.1074/jbc.M111.324012 PMC3318738 22247546

[19] Provencher EAP, Ehrig MR, Cecere AG, Cousins SC, Maybin MA, Meredith TC, Miyashiro TI. 2023. Inhibition of biofilm formation by a lipopolysaccharide-associated glycosyltransferase in the bacterial symbiont *Vibrio fischeri**.* Front Bacteriol 2. doi:10.3389/fbrio.2023.1254305

[20] Zhu S, Kojima S, Homma M. 2013. Structure, gene regulation and environmental response of flagella in *Vibrio*. Front Microbiol 4:410. doi:10.3389/fmicb.2013.00410 PMC3872333 24400002

[21] Dufrêne YF, Persat A. 2020. Mechanomicrobiology: how bacteria sense and respond to forces. Nat Rev Microbiol 18:227–240. doi:10.1038/s41579-019-0314-2 31959911

[22] Pollack-Berti A, Wollenberg MS, Ruby EG. 2010. Natural transformation of *Vibrio fischeri* requires *tfoX* and *tfoY*. Environ Microbiol 12:2302–2311. doi:10.1111/j.1462-2920.2010.02250.x PMC3034104 21966921

[23] Gifford SM, Sharma S, Rinta-Kanto JM, Moran MA. 2011. Quantitative analysis of a deeply sequenced marine microbial metatranscriptome. ISME J 5:461–472. doi:10.1038/ismej.2010.141 PMC3105723 20844569

[24] Gifford SM, Becker JW, Sosa OA, Repeta DJ, DeLong E. 2016. Quantitative transcriptomics reveals the growth- and nutrient-dependent response of a streamlined marine methylotroph to methanol and naturally occurring dissolved organic matter. mBio 7:e01279-16. doi:10.1128/mBio.01279-16 PMC5120137 27879330

[25] Neidhart FC, Curtiss R. 1996. Escherichia coli and Salmonella typhimurium: cellular and molecular biology, p 1553–1569. American Society for Microbiology, Washington, DC.

[26] Taniguchi Y, Choi PJ, Li G-W, Chen H, Babu M, Hearn J, Emili A, Xie XS. 2010. Quantifying *E. coli* proteome and transcriptome with single-molecule sensitivity in single cells. Science 329:533–538. doi:10.1126/science.1188308 PMC2922915 20671182

[27] Rendueles O. 2020. Deciphering the role of the capsule of *Klebsiella pneumoniae* during pathogenesis: a cautionary tale. Mol Microbiol 113:883–888. doi:10.1111/mmi.14474 PMC7317218 31997409

[28] Millikan DS, Ruby EG. 2003. FlrA, a σ^54^-dependent transcriptional activator in *Vibrio fischeri*, is required for motility and symbiotic light-organ colonization. J Bacteriol 185:3547–3557. doi:10.1128/JB.185.12.3547-3557.2003 PMC156232 12775692

[29] Deloney-Marino CR, Visick KL. 2012. Role for *cheR* of *Vibrio fischeri* in the *Vibrio*-squid symbiosis. Can J Microbiol 58:29–38. doi:10.1139/w11-107 PMC3256272 22182211

[30] Mandel MJ, Schaefer AL, Brennan CA, Heath-Heckman EAC, Deloney-Marino CR, McFall-Ngai MJ, Ruby EG. 2012. Squid-derived chitin oligosaccharides are a chemotactic signal during colonization by *Vibrio fischeri*. Appl Environ Microbiol 78:4620–4626. doi:10.1128/AEM.00377-12 PMC3370474 22522684

[31] Brennan CA, Mandel MJ, Gyllborg MC, Thomasgard KA, Ruby EG. 2013. Genetic determinants of swimming motility in the squid light-organ symbiont *Vibrio fischeri**.* Microbiologyopen 2:576–594. doi:10.1002/mbo3.96 PMC3948606 23907990

[32] McFall-Ngai M, Brennan C, Weis V, Lamarcq L. 1998. Mannose adhesin—glycan interactions in the *Euprymna scolopes*—*Vibrio fischeri* symbiosis, p 273–276. *In* Gal YL, Halvorson HO (ed), New developments in marine biotechnology. Springer US, Boston, MA.

[33] Miyashiro T, Klein W, Oehlert D, Cao X, Schwartzman J, Ruby EG. 2011. The N-acetyl-D-glucosamine repressor NagC of *Vibrio fischeri* facilitates colonization of *Euprymna scolopes*. Mol Microbiol 82:894–903. doi:10.1111/j.1365-2958.2011.07858.x PMC3212624 21992506

[34] Graf J, Ruby EG. 1998. Host-derived amino acids support the proliferation of symbiotic bacteria. Proc Natl Acad Sci USA 95:1818–1822. doi:10.1073/pnas.95.4.1818 PMC19196 9465100

[35] Septer AN, Wang Y, Ruby EG, Stabb EV, Dunn AK. 2011. The haem-uptake gene cluster in *Vibrio fischeri* is regulated by Fur and contributes to symbiotic colonization. Environ Microbiol 13:2855–2864. doi:10.1111/j.1462-2920.2011.02558.x PMC4000482 21883801

[36] Graf J, Ruby EG. 2000. Novel effects of a transposon insertion in the *Vibrio fischeri glnD* gene: defects in iron uptake and symbiotic persistence in addition to nitrogen utilization. Mol Microbiol 37:168–179. doi:10.1046/j.1365-2958.2000.01984.x 10931314

[37] Eickhoff MJ, Bassler BL. 2020. *Vibrio fischeri siderophore* production drives competitive exclusion during dual-species growth. Mol Microbiol 114:244–261. doi:10.1111/mmi.14509 PMC7541421 32259318

[38] Wang Y, Dufour YS, Carlson HK, Donohue TJ, Marletta MA, Ruby EG. 2010. H-NOX–mediated nitric oxide sensing modulates symbiotic colonization by *Vibrio fischeri*. Proc Natl Acad Sci USA 107:8375–8380. doi:10.1073/pnas.1003571107 PMC2889544 20404170

[39] Dunn AK. 2012. *Vibrio fischeri* metabolism: symbiosis and beyond. Adv Microb Physiol 61:37–68. doi:10.1016/B978-0-12-394423-8.00002-0 23046951

[40] Dunn AK. 2018. Alternative oxidase activity reduces stress in *Vibrio fischeri* cells exposed to nitric oxide. J Bacteriol 200:e00797-17. doi:10.1128/JB.00797-17 PMC6040193 29760206

[41] Dunn A. K. 2023. Alternative oxidase in bacteria. Biochim Biophys Acta 1864:148929. doi:10.1016/j.bbabio.2022.148929 36265564

[42] Dunn Anne K, Karr EA, Wang Y, Batton AR, Ruby EG, Stabb EV. 2010. The alternative oxidase (AOX) gene in *Vibrio fischeri* is controlled by NsrR and upregulated in response to nitric oxide. Mol Microbiol 77:44–55. doi:10.1111/j.1365-2958.2010.07194.x PMC3682826 20487270

[43] Shimizu T, Matsumoto A, Noda M. 2019. Cooperative roles of nitric oxide-metabolizing enzymes to counteract nitrosative stress in enterohemorrhagic *Escherichia coli*. Infect Immun 87:e00334-19. doi:10.1128/IAI.00334-19 PMC6704613 31209149

[44] Upadhyay AK, Hooper AB, Hendrich MP. 2006. NO reductase activity of the tetraheme cytochrome C554 of *Nitrosomonas europaea**.* J Am Chem Soc 128:4330–4337. doi:10.1021/ja055183+ PMC2806813 16569009

[45] Wang J, Vine CE, Balasiny BK, Rizk J, Bradley CL, Tinajero-Trejo M, Poole RK, Bergaust LL, Bakken LR, Cole JA. 2016. The roles of the hybrid cluster protein, Hcp and its reductase, Hcr, in high affinity nitric oxide reduction that protects anaerobic cultures of *Escherichia coli* against nitrosative stress. Mol Microbiol 100:877–892. doi:10.1111/mmi.13356 26879449

[46] Wang Y, Dunn AK, Wilneff J, McFall-Ngai MJ, Spiro S, Ruby EG. 2010. *Vibrio fischeri* flavohaemoglobin protects against nitric oxide during initiation of the squid-*Vibrio* symbiosis. Mol Microbiol 78:903–915. doi:10.1111/j.1365-2958.2010.07376.x PMC2978254 20815823

[47] Guckes KR, Cecere AG, Williams AL, McNeil AE, Miyashiro T. 2020. The bacterial enhancer binding protein vash promotes expression of a type VI secretion system in *Vibrio fischeri* during symbiosis. J Bacteriol 202:e00777–00719. doi:10.1128/JB.00777-19 PMC7167466 31964698

[48] Shao X, Zhang X, Zhang Y, Zhu M, Yang P, Yuan J, Xie Y, Zhou T, Wang W, Chen S, Liang H, Deng X. 2018. RpoN-dependent direct regulation of quorum sensing and the type VI secretion system in *Pseudomonas aeruginosa* PAO1. J Bacteriol 200:e00205–00218. doi:10.1128/JB.00205-18 PMC6060356 29760208

[49] Guan J, Xiao X, Xu S, Gao F, Wang J, Wang T, Song Y, Pan J, Shen X, Wang Y. 2015. Roles of RpoS in *Yersinia pseudotuberculosis* stress survival, motility, biofilm formation and type VI secretion system expression. J Microbiol 53:633–642. doi:10.1007/s12275-015-0099-6 26310305

[50] Bouteiller M, Gallique M, Bourigault Y, Kosta A, Hardouin J, Massier S, Konto-Ghiorghi Y, Barbey C, Latour X, Chane A, Feuilloley M, Merieau A. 2020. Crosstalk between the type VI secretion system and the expression of class IV flagellar genes in the *Pseudomonas fluorescens* MFE01 strain. Microorganisms 8:622. doi:10.3390/microorganisms8050622 PMC7286023 32344878

[51] Liu L, Hao S, Lan R, Wang G, Xiao D, Sun H, Xu J. 2015. The type VI secretion system modulates flagellar gene expression and secretion in *Citrobacter freundii* and contributes to adhesion and cytotoxicity to host cells. Infect Immun 83:2596–2604. doi:10.1128/IAI.03071-14 PMC4468558 25870231

[52] Lele PP, Hosu BG, Berg HC. 2013. Dynamics of mechanosensing in the bacterial flagellar motor. Proc Natl Acad Sci USA 110:11839–11844. doi:10.1073/pnas.1305885110 PMC3718179 23818629

[53] Chawla R, Ford KM, Lele PP. 2017. Torque, but not FliL, regulates mechanosensitive flagellar motor-function. Sci Rep 7:5565. doi:10.1038/s41598-017-05521-8 PMC5514156 28717192

[54] Belas R. 2014. Biofilms, flagella, and mechanosensing of surfaces by bacteria. Trends Microbiol 22:517–527. doi:10.1016/j.tim.2014.05.002 24894628

[55] Belas R, Simon M, Silverman M. 1986. Regulation of lateral flagella gene transcription in *Vibrio parahaemolyticus*. J Bacteriol 167:210–218. doi:10.1128/jb.167.1.210-218.1986 PMC212863 3013835

[56] Kawagishi I, Imagawa M, Imae Y, McCarter L, Homma M. 1996. The sodium-driven polar flagellar motor of marine *Vibrio* as the mechanosensor that regulates lateral flagellar expression. Mol Microbiol 20:693–699. doi:10.1111/j.1365-2958.1996.tb02509.x 8793868

[57] Gode-Potratz CJ, Kustusch RJ, Breheny PJ, Weiss DS, McCarter LL. 2011. Surface sensing in *Vibrio parahaemolyticus* triggers a programme of gene expression that promotes colonization and virulence. Mol Microbiol 79:240–263. doi:10.1111/j.1365-2958.2010.07445.x PMC3075615 21166906

[58] Salomon D, Gonzalez H, Updegraff BL, Orth K. 2013. *Vibrio parahaemolyticus* type VI secretion system 1 is activated in marine conditions to target bacteria, and is differentially regulated from system 2. PLoS One 8:e61086. doi:10.1371/journal.pone.0061086 PMC3628861 23613791

[59] López CM, Rholl DA, Trunck LA, Schweizer HP. 2009. Versatile dual-technology system for markerless allele replacement in *Burkholderia pseudomallei**.* Appl Environ Microbiol 75:6496–6503. doi:10.1128/AEM.01669-09 PMC2765137 19700544

[60] Speare L, Septer AN. 2019. Coincubation assay for quantifying competitive interactions between *Vibrio fischeri* isolates. J Vis Exp 149. doi:10.3791/59759 31380831

[61] Smith S, Salvato F, Garikipati A, Kleiner M, Septer AN. 2021. Activation of the Type VI secretion system in the squid symbiont *Vibrio fischeri* requires the transcriptional regulator TasR and the structural proteins TssM and TssA. J Bacteriol 203. doi:10.1128/JB.00399-21 PMC8508121 34370559

[62] van den Belt M, Gilchrist C, Booth TJ, Chooi Y-H, Medema MH, Alanjary M. 2023. CAGECAT: the CompArative GEne cluster analysis toolbox for rapid search and visualisation of homologous gene clusters. BMC Bioinformatics 24:181. doi:10.1186/s12859-023-05311-2 PMC10155394 37131131

[63] Gilchrist CLM, Chooi Y-H. 2021. Clinker & clustermap.js: automatic generation of gene cluster comparison figures. Bioinformatics 37:2473–2475. doi:10.1093/bioinformatics/btab007 33459763

[64] Bolger AM, Lohse M, Usadel B. 2014. Trimmomatic: a flexible trimmer for Illumina sequence data. Bioinformatics 30:2114–2120. doi:10.1093/bioinformatics/btu170 PMC4103590 24695404

[65] Langmead B, Trapnell C, Pop M, Salzberg SL. 2009. Ultrafast and memory-efficient alignment of short DNA sequences to the human genome. Genome Biol 10:1–10. doi:10.1186/gb-2009-10-3-r25 PMC2690996 19261174

[66] Anders S, Pyl PT, Huber W. 2015. HTSeq—a Python framework to work with high-throughput sequencing data. Bioinformatics 31:166–169. doi:10.1093/bioinformatics/btu638 PMC4287950 25260700

[67] Dial CN, Speare L, Sharpe GC, Gifford SM, Septer AN, Visick KL. 2021. Para-aminobenzoic acid, calcium, and c-di-GMP induce formation of cohesive, Syp-polysaccharide-dependent biofilms in *Vibrio fischeri**.* mBio 12:e0203421. doi:10.1128/mBio.02034-21 PMC8546588 34607467

[68] Oksanen J, Simpson GL, Blanchet FG, Kindt R, Legendre P, Minchin PR, O’Hara RB, Solymos P, Stevens MHH, Szoecs E. 2013. Vegan: community ecology package. R Package Version. 2.0-10. CRAN.

